# Fixation of a Felix IV Periprosthetic Tibia Fracture Using Synthetic Ligaments: A Case Report

**DOI:** 10.7759/cureus.96147

**Published:** 2025-11-05

**Authors:** Ahmed Shalaan, Manoj Sood, Dan Arvinte

**Affiliations:** 1 Trauma and Orthopaedics, Bedfordshire Hospitals NHS Foundation Trust, Bedford, GBR

**Keywords:** arthroplasty, case report, fiberwire, fracture, knee, ortho-tape, periprosthetic, polyethylene terephthalate neoligaments

## Abstract

Fixation of the Felix IV tibial fractures after total knee arthroplasty is complicated. Additionally, the rarity of this type of injury further complicates the situation due to the lack of established fixation techniques, in contrast to other periprosthetic knee fractures. Artificial ligaments are currently used in a wide range of orthopedic procedures, from anterior cruciate ligament reconstruction to tendon and ligament augmentation in different joints. Using artificial ligaments for these specific types of fractures may offer an advantage.

A lady in her late 70s presented with a Felix IV fracture, ongoing knee pain, and distal wound dehiscence following a twisting knee injury on postoperative day 14 after her complex primary total knee replacement, where a rotating hinge implant was used. In this particular case, fixation with plates and screws was not possible due to poor bone quality and the nature of the implant. Therefore, fixation was performed using Ortho-tape, a polyethylene terephthalate (polyester) (Neoligaments, Leeds, United Kingdom), along with FiberWire (Ethicon, Raritan, New Jersey, United States) to manage her injury.

The aim of this report is to introduce a novel surgical technique employing artificial ligaments, specifically Ortho-tape, a polyethylene terephthalate (polyester) material (Neoligaments), and FiberWire (Ethicon), without the use of metal components. Positive clinical and radiographic outcomes at the 18-month follow-up demonstrate that this method is effective in restoring knee joint extension, minimizing disabilities, and alleviating pain associated with periprosthetic Felix IV fractures.

## Introduction

The incidence of periprosthetic fractures following primary total knee arthroplasty ranges from 0.3% to 2.5%, while for revision total knee arthroplasty, it is between 1.6% and 38%. Supracondylar femoral fractures are the most common periprosthetic fracture, occurring in 0.3-2.5% of cases. Patellar fractures follow, with an incidence of 0.2-21%, and tibial fractures occur in 0.4-1.7% of cases [1-3].

Although most of these fractures are a result of low-energy trauma or non-traumatic events, some may happen following a fall, major trauma, stress fractures, or a non-united tibial tuberosity osteotomy (TTO) after revision surgeries. Key risk factors include advanced age (>70 years), female gender, diabetes, osteoporosis, rheumatoid arthritis, chronic corticosteroid use, and revision surgeries [4-8].

Treatment options for periprosthetic tibial tuberosity fractures (Felix IV) range from conservative management for undisplaced fractures to surgical procedures such as open reduction internal fixation using lag screws alone, plate and screw fixation, tension band wiring, allograft or autograft reconstruction, or synthetic attachment tube [9-12]. The uncommon nature of these fractures, combined with the diversity of the fracture patterns and patient factors, complicates the identification of a universally successful treatment method.

This study presents a case involving the reconstruction of the knee joint's extension system using patellar tape, a polyethylene terephthalate (polyester) material (Neoligaments, Leeds, United Kingdom), and FiberWire (Ethicon, Raritan, New Jersey, United States), following a periprosthetic Felix IV fracture. To the best of our knowledge, this is the first reported application of this technique for this type of fracture.

## Case presentation

Past medical history 

The patient was a lady in her late 70s with multiple comorbidities, including osteoporosis, cardiac disease requiring a pacemaker, and a prior stroke, for which she was on many medications, including aspirin. She was a non-smoker and non-drinker, with a BMI of 21. Despite assistance with a Zimmer frame, she was able to walk only short distances.

Initial surgery 

**Figure 1:**
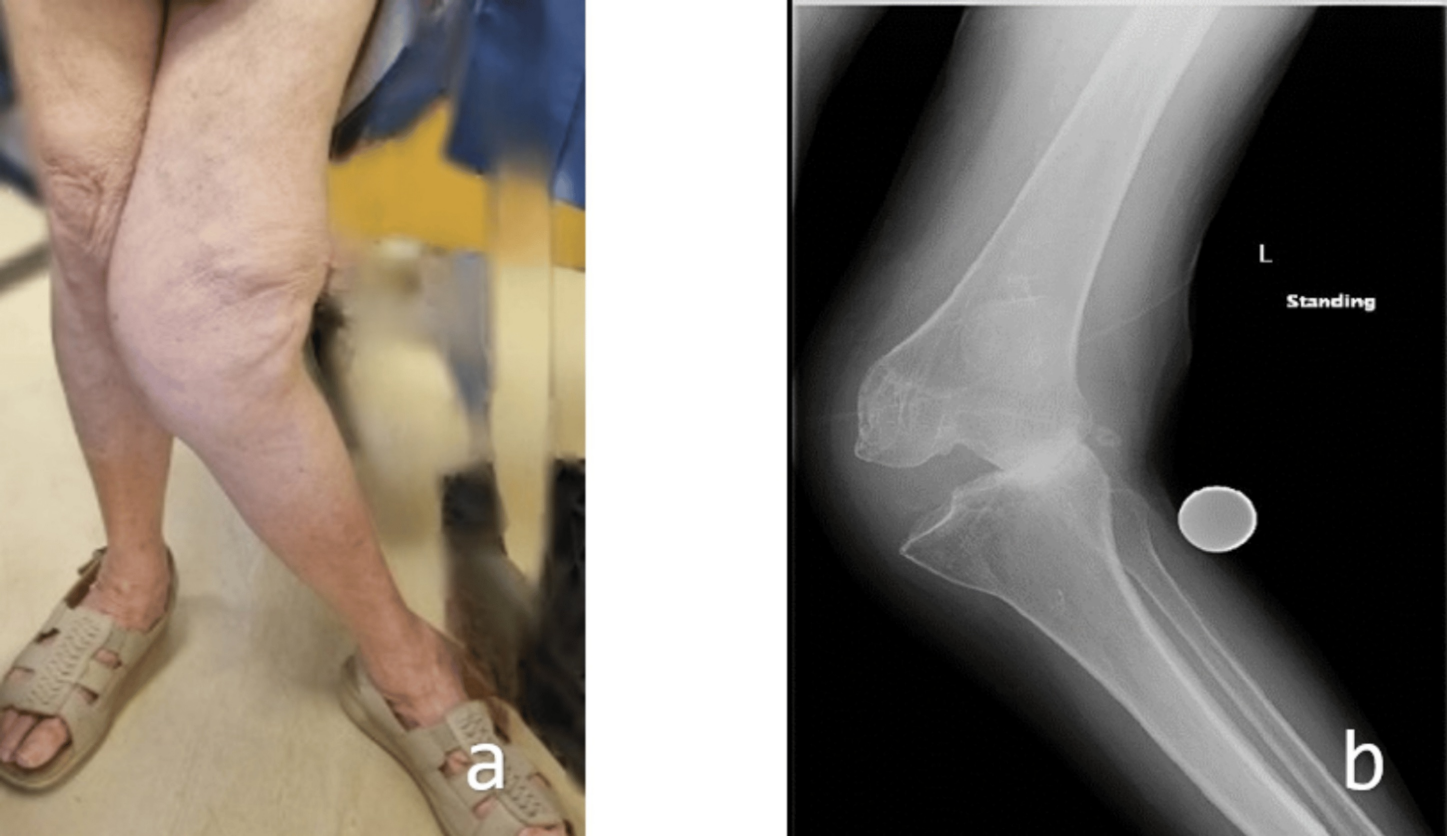
Severe preoperative valgus deformity clinically (a) and radiologically (b)

She underwent a hemiarthroplasty for a right neck of femur fracture. This resulted in a shorter right lower limb, creating a limb length discrepancy that further impaired her mobility. The altered gait mechanics aggravated her pre-existing left knee osteoarthritis and deformity. Two months after, her left knee X-rays showed advanced osteoarthritis with severe valgus deformity. Clinically, she had an evident valgus gait thrust and an extension lag of approximately 10°, with flexion limited to 100°. Radiographic assessment confirmed a 30° valgus deformity, which was found to be partially correctable (Figure 1).

Complex primary total knee replacement

**Figure 2:**
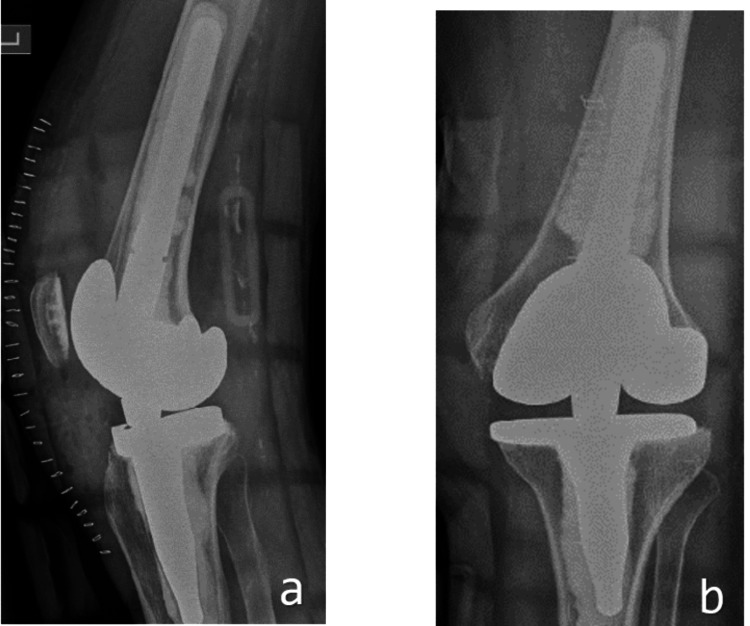
Postoperative anteroposterior (a) and lateral (b) X-rays showing rotating hinge (NexGen, Zimmer) total knee replacement with deformity correction

Later, about six months after her hip surgery, she underwent a complex total knee replacement with a hinged rotating platform. The surgery revealed an incompetent medial collateral ligament (MCL) and tight lateral structures. We used a rotating hinge knee (NexGen, Zimmer, Warsaw, Indiana, United States) with a distal femoral cut of 12 mm and a tibial cut of 6 mm from the medial side, using a size D femoral component and a size 3 tibial component with a size 12 insert. Postoperatively, her X-rays were satisfactory, and her range of motion was from 0° to 110° (Figure 2).

Complication and management

Approximately two weeks postoperative, while still undergoing rehabilitation, the patient sustained a twisting knee injury. Although the twist was not severe, it resulted in an avulsion injury of the tibial tuberosity with distal wound dehiscence, measuring 5 cm in length, 4 cm in width, and 3 cm in depth. Consequently, she was taken back to the theatres for fracture fixation using patellar tape, a polyethylene terephthalate (polyester) material (Neoligaments), and FiberWire (Ethicon). The tuberosity was temporarily fixed with a 1.6 mm K-wire, and a tunnel was drilled anterior to the tibial stem and distal to the tibial tuberosity fracture, at the point where the tibial width allows maximum purchase to reduce the risk of pull-out or cortical breach. The patellar tape (Neoligaments) was utilized in a figure-of-eight configuration, passing around the patella proximally and through the tibial tunnel distally, with the crossing positioned over the avulsed tuberosity to enhance the compression around the fracture site. Tensioning was performed with the knee in approximately 30° of flexion to optimize stability and tension.

**Figure 3:**
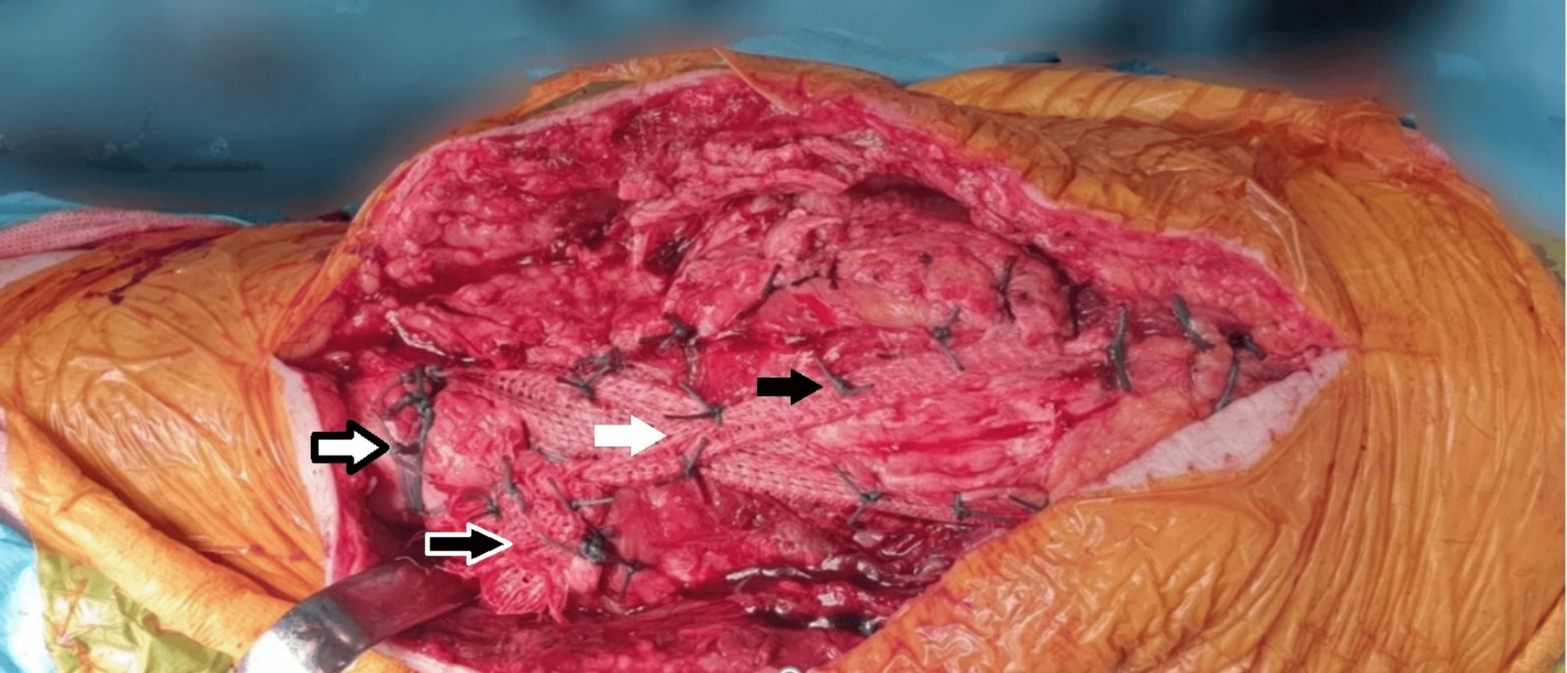
Intraoperative image showing the patellar tape (Neoligaments) was utilized in a figure-of-eight configuration reinforced with number 5 FiberWire (Ethicon) sutures Intraoperative image showing the patellar tape (Neoligaments) passing through the tibial tunnel (white arrow with black outline) in a figure-of-eight configuration, with the crossing positioned over the tibial tuberosity fracture (white arrow) to enhance compression. The knot (black arrow with white outline) should always be on the lateral side of the construct to ensure adequate soft tissue coverage. The construct was reinforced with number 5 FiberWire (Ethicon) sutures (black arrow).

To reinforce the repair, two number 5 FiberWire (Ethicon) sutures were applied circumferentially around the patellar tape (Neoligaments), keeping the tape as flat as possible to avoid tape roll-over or the formation of bumps and to maintain even compression at the fracture site (Figure 3). The overall technique functioned as a tension band principle, converting the distraction force into a compression force. It is worth mentioning that the knot should always be positioned laterally to ensure adequate soft tissue coverage. Postoperatively, the knee was immobilized in a knee immobilizer locked in full extension, and the X-rays showed good reduction of the fracture. Antibiotic coverage was provided for 24 hours, and the patient was instructed to bear weight as tolerated, being safely discharged after 10 days.

Early follow-ups 

At the first follow-up appointment, six weeks after the operation, the patient exhibited satisfactory wound healing. Her active range of motion was 10° to 100°, with an extension lag of approximately 10°. Recurrent falls were noted, mainly due to contralateral leg shortening secondary to her previous hip hemiarthroplasty. Though mild proximal migration of the tibial tuberosity was evident on her X-rays, there was no significant knee injury.

The patient was readmitted twice afterwards due to these recurrent falls. She was referred to Orthotics, and though her limb length discrepancy was deemed too substantial for complete compensation, the device noticeably improved her mobility and stability. At the later follow-up appointment, her extension lag slightly worsened to 20°. The X-rays showed some proximal migration of the tibial tuberosity fragment from 2 mm at her first visit (six weeks postoperative) to 5 mm 12 weeks postoperative, with good callus formation.

Late follow-up 

**Figure 4:**
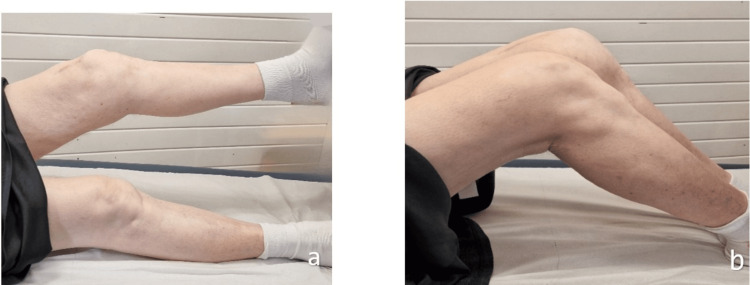
Clinical follow-up 18 months postoperative showing intact straight leg raising test (a) and good range of motion and limb length discrepancy (b)

**Figure 5:**
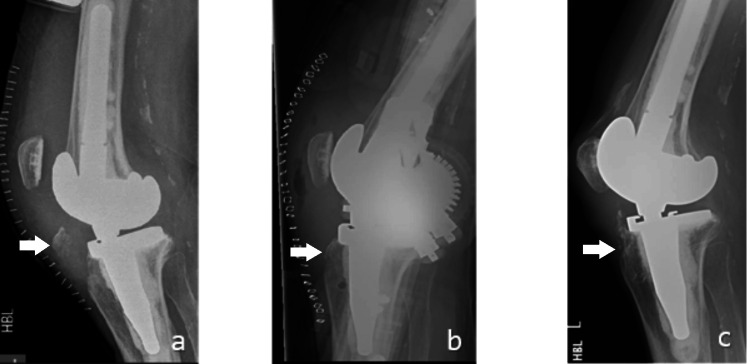
Serial lateral knee X-rays showing fracture tibial tuberosity (a), postoperative day 1 after fixation showing the successful reduction of the fracture (b), and postoperative after 18 month showing minimal proximal migration and good callus formation (c) Serial lateral knee radiographs: preoperative image showing tibial tuberosity fracture (arrow indicates fractured tuberosity) (a), postoperative day 1 image showing reduction and fixation (arrow indicates the fixation construct) (b), and 18-month postoperative image showing minimal proximal migration and good callus formation (arrow indicates united fracture) (c).

At her final follow-up, 18 months after the second surgery, her mobility remained limited, partly due to prior medical conditions, including a recent diagnosis of dementia. She also had a true limb length discrepancy of 2 inches, which was partially corrected with a custom-built right shoe. Despite these limitations, she exhibited an active range of knee motion from 20° to 90° flexion and could perform a straight leg raise test, although with a 20° extension lag (Figure 4). Follow-up X-rays revealed no further displacement of the tibial tuberosity and showed increased callus formation around the fracture site (Figure 5).

## Discussion

Periprosthetic fractures occurring in total knee arthroplasty represent a growing concern for orthopedic surgeons. According to the National Joint Registry (NJR) for the United Kingdom 2023 report, these fractures account for 5% of revision surgeries, ranking eighth after aseptic loosening (37.5%), instability (17.2%), implant wear (14%), pain (13.7%), other indications (10.8%), infection (8.4%), and malalignment (7.1%) [13]. For elderly patients, early postoperative mobilization is crucial to prevent the health complications associated with immobility. However, achieving primary stability that allows full weight-bearing, although desirable, is not always possible after fixation of the fracture. In most cases, partial weight-bearing is recommended with the assistance of frames [10,14].

Type IV fractures are rare, and there is no consensus regarding their optimal treatment strategy. The most widely accepted classification, proposed by Felix et al. in 1997, categorizes these fractures based on their position relative to the tibial component and the stability of the component. There are four types: type I occurs through the tibial plateau, type II occurs below the tibial plateau adjacent to the tibial stem, type III occurs distal to the tibial stem, and type IV involves the tibial tubercle. Each type has three subtypes based on stability: A (stable implant), B (loose implant), and C (intraoperative fractures). In that study, 102 proximal tibia periprosthetic fractures were included. Only two of them were type IV: one was treated with screw fixation, while the other was treated with successful cast immobilization [6,12]. Liu et al. reported that Felix IV fractures exhibited the shortest healing time compared to types II and III. It is worth mentioning that they had only a single case of Felix IV A that required subsequent hardware removal due to stiffness [15].

Treating periprosthetic tibial fractures is challenging due to the interference from the tibial stem. Type IV fractures, in particular, require special attention due to the disruption of the extensor mechanism. Treatment options for Felix IV periprosthetic fractures include conservative management or open reduction internal fixation using various methods such as cannulated screws, plates and screws, or even tension band wiring. Hanssen and Stuart proposed the use of polypropylene mesh tape or rerouting the semitendinosus tendon for the fixation of type IV tibial fractures [6].

Furthermore, techniques involving attachment tubes for the synthetic reconstruction of the knee joint's extensor mechanism have shown promising results. First described by Browne and Hanssen in 2011 [11], these techniques have been utilized by others to treat Felix IV fractures, revise failed Felix IV fixations, and manage other causes of extensor mechanism disruption following total knee arthroplasty [5,16]. Richter et al. used a similar technique to revise a non-united Felix IV fracture fixed with a third tubular plate, employing a polyethylene terephthalate attachment tube. This technique produced better outcomes compared to fixation with plates and screws [9].

In our technique, we utilized Ortho-tape, a polyethylene terephthalate (polyester) material (Neoligaments), along with FiberWire (Ethicon), which provided a novel and effective solution for fracture fixation and stabilization. To our knowledge, this is the first report using this technique. While the results were encouraging, the fixation was not completely rigid, as a 5 mm proximal migration was observed in a later follow-up. The patient's limb length discrepancy and general frailty also affected recovery, making it difficult to separate the effect of the technique from her overall condition. Furthermore, as this is only a single case, broader conclusions cannot yet be drawn.

Nevertheless, the construct operated as a tension band, reducing strain on the prosthesis and the fracture site, thereby minimizing the risk of failure. This combination of innovative techniques offered several additional advantages. Firstly, it provided sufficient stability for early mobilization, facilitating postoperative rehabilitation and reducing the risk of joint stiffness. Secondly, it decreased the load transferred to the fracture site by converting tension force to compression force, promoting bone healing and preventing implant-related complications. Thirdly, by utilizing this technique, we avoided the use of metalwork in this area, potentially preventing wound complications and inconvenience for some patients who might require subsequent metalwork removal. Lastly, the use of non-absorbable materials ensured the long-term durability of the construct, reducing the likelihood of revision surgery.

## Conclusions

The management of Felix IV fractures is challenging due to the complex fracture pattern, the underlying prosthesis, and the patient's frailty, often leading to complications such as pain, extensor mechanism dysfunction, and prominent metalwork. We here present a new surgical technique to treat these fractures using an artificial ligament (Ortho-tape, Neoligaments) plus FiberWire (Ethicon), entirely avoiding any metalwork. Positive clinical and radiologic results on follow-up after 18 months indicated that the use of this technique effectively restores knee joint extension and reduces knee pain after periprosthetic Felix IV (tibial tuberosity) fracture. Given the rarity of these fractures, future studies, including biomechanical testing and larger clinical series, are needed to validate the strength, reproducibility, and long-term durability of this approach.
